# Risk prediction of heart failure in patients with ischemic heart disease using network analytics and stacking ensemble learning

**DOI:** 10.1186/s12911-023-02196-2

**Published:** 2023-05-23

**Authors:** Dejia Zhou, Hang Qiu, Liya Wang, Minghui Shen

**Affiliations:** 1 School of Computer Science and Engineering, University of Electronic Science and Technology of China, No.2006, Xiyuan Ave, West Hi-Tech Zone, Chengdu, Sichuan 611731 P.R. China; 2 Big Data Research Center, University of Electronic Science and Technology of China, Chengdu, China; 3 Health Information Center of Sichuan Province, Chengdu, China

**Keywords:** Administrative data, Ischemic heart disease, Heart failure, Comorbidity network, Network feature, Ensemble learning

## Abstract

**Background:**

Heart failure (HF) is a major complication following ischemic heart disease (IHD) and it adversely affects the outcome. Early prediction of HF risk in patients with IHD is beneficial for timely intervention and for reducing disease burden.

**Methods:**

Two cohorts, cases for patients first diagnosed with IHD and then with HF (N = 11,862) and control IHD patients without HF (N = 25,652), were established from the hospital discharge records in Sichuan, China during 2015-2019. Directed personal disease network (PDN) was constructed for each patient, and then these PDNs were merged to generate the baseline disease network (BDN) for the two cohorts, respectively, which identifies the health trajectories of patients and the complex progression patterns. The differences between the BDNs of the two cohort was represented as disease-specific network (DSN). Three novel network features were exacted from PDN and DSN to represent the similarity of disease patterns and specificity trends from IHD to HF. A stacking-based ensemble model DXLR was proposed to predict HF risk in IHD patients using the novel network features and basic demographic features (i.e., age and sex). The Shapley Addictive exPlanations method was applied to analyze the feature importance of the DXLR model.

**Results:**

Compared with the six traditional machine learning models, our DXLR model exhibited the highest AUC (0.934 ± 0.004), accuracy (0.857 ± 0.007), precision (0.723 ± 0.014), recall (0.892 ± 0.012) and F_1_ score (0.798 ± 0.010). The feature importance showed that the novel network features ranked as the top three features, playing a notable role in predicting HF risk of IHD patient. The feature comparison experiment also indicated that our novel network features were superior to those proposed by the state-of-the-art study in improving the performance of the prediction model, with an increase in AUC by 19.9%, in accuracy by 18.7%, in precision by 30.7%, in recall by 37.4%, and in F_1_ score by 33.7%.

**Conclusions:**

Our proposed approach that combines network analytics and ensemble learning effectively predicts HF risk in patients with IHD. This highlights the potential value of network-based machine learning in disease risk prediction field using administrative data.

## Background

Ischemic heart disease (IHD) is one of the major underlying causes of heart failure (HF) [1–3] and is related to increase mortality [4, 5]. Abdissa et al. [6] investigated 306 IHD patients, 64.1% of whom developed HF with the number of females being about twice the number of males. In our previous study on the comorbidity patterns of IHD patients, HF occurred in 29.39% of the IHD patients, and the incidence of HF in IHD patients was eight times higher than that in patients without IHD [7]. HF, as a complex cardiovascular syndrome, causes frequent hospitalization, leads to low quality of life, and accounts for a large portion of cardiovascular disease (CVD) morbidity and mortality [8, 9]. Therefore, early prediction of HF risk in IHD patients may improve patients’ outcomes, and reduce medical costs and mortality.

In recent years, as a sub-filed of artificial intelligence, machine learning (ML) techniques attracted much attention in the medical domain [10–17], and have been increasingly employed for HF prediction [18–25]. For instance, Rammal et al. [26] integrated different types of data, including demographic data, chest X-ray images data, and clinical diagnostic and symptoms data, of 100 HF patients to construct random forest (RF) and logistic regression (LR) predictive models which both achieved an accuracy of 93%. Akbilgic et al. [27] developed a convolutional neural network (CNN) model that utilized electrocardiographic (ECG) data for predicting the risk of developing HF, which achieved an AUC of 75%. According to Chen et al. [28], they employed demographic data, diagnostic data, clinical test data and intraoperative monitoring data of patients to construct a model with deep pyramid CNN and extreme gradient boosting (XGBoost) method for forecasting the risk of HF after operation. Although the recent advances in ML techniques have significantly improved the prediction accuracy for HF, these works have two major issues. First, most ML models developed so far relied on multiple types of detailed medical data, such as cardiac image, laboratory examination and ECG data, and these models mainly aimed to interpret clinical data and assist clinicians for screening and diagnosis of HF. Few studies have attempted to establish ML-based risk prediction models for people who are likely to progress to HF (e.g., patients with IHD) when detailed diagnostic tests are unavailable. Second, these methods considered comorbidities, such as hypertension, diabetes and atrial fibrillation, as clinical risk factors for HF, but did not consider their complex relationships and progression patterns among comorbidities. Since HF is a major complication following IHD, capturing the disease progression pathways can reveal the multimorbidity risk, thus increasing the accuracy of disease risk prediction.

Recently, the availability of large amounts of administrative data (e.g., hospital discharge records, HDR) and the development of network theory provide new opportunities to apply a predictive model for improving the disease risk assessment. The administrative data contains useful proxies for missing clinical predictors, e.g. diagnoses and procedures recorded during hospitalizations, and the network analysis offers effective approach to explore comorbidity patterns [29–31], and the temporal disease trajectories [32–34] of patients hidden in these data. For instance, a cross-sectional study [35] used electronic health records of 34,099 discharged patients and network analysis techniques and determined that the comorbidity networks of CVDs were highly centralized in prevalent diseases, such as cardiac arrhythmias, HF, chronic kidney disease, hypertension, and ischemic diseases. Using large-scale datasets and network science, Ong et al. [36] constructed a directed disease network to identify rare and novel disease patterns in pediatric pulmonary hypertension. Nevertheless, to the best of our knowledge, only very few studies have further combined network analytics with ML techniques to improve the healthcare system [37–40], especially for disease risk prediction. Khan et al. [41] constructed disease networks from 1.4 million admission records to predict the risk of type 2 diabetes. Their results showed that the measurements based on network theory ranked highest among the parameter estimation, LR, and decision tree (DT) models, with 82–87% prediction accuracy. Hossain et al. [37] developed risk prediction models using social network analysis on administrative datasets to determine the risk of type 2 diabetes in patients with CVD. They extracted three network-based features from the comorbidity network to indicate the comorbidity prevalence, transfer pattern, and cluster membership, and they constructed ML models with 79–88% accuracy. Using network analytics and administrative data, Uddin et al. [42] constructed five traditional ML models and two deep learning models to predict the number of chronic diseases. They concluded that the network analysis approach allowed them to better represent the relationship among patients’ diseases.

This study aims to identify IHD patients at high risk of HF. Inspired by the previous studies [37, 41], we propose a risk prediction approach using disease network analysis combined with ensemble learning technique based on routinely collected administrative data. It has two goals: (1) design novel network features to capture the specific complex progression pattern from IHD to HF; (2) develop a stacking-based ensemble model to predict HF risk for IHD patients using basic demographic information and network features.

## Methods

### Overview

**Fig. 1:**
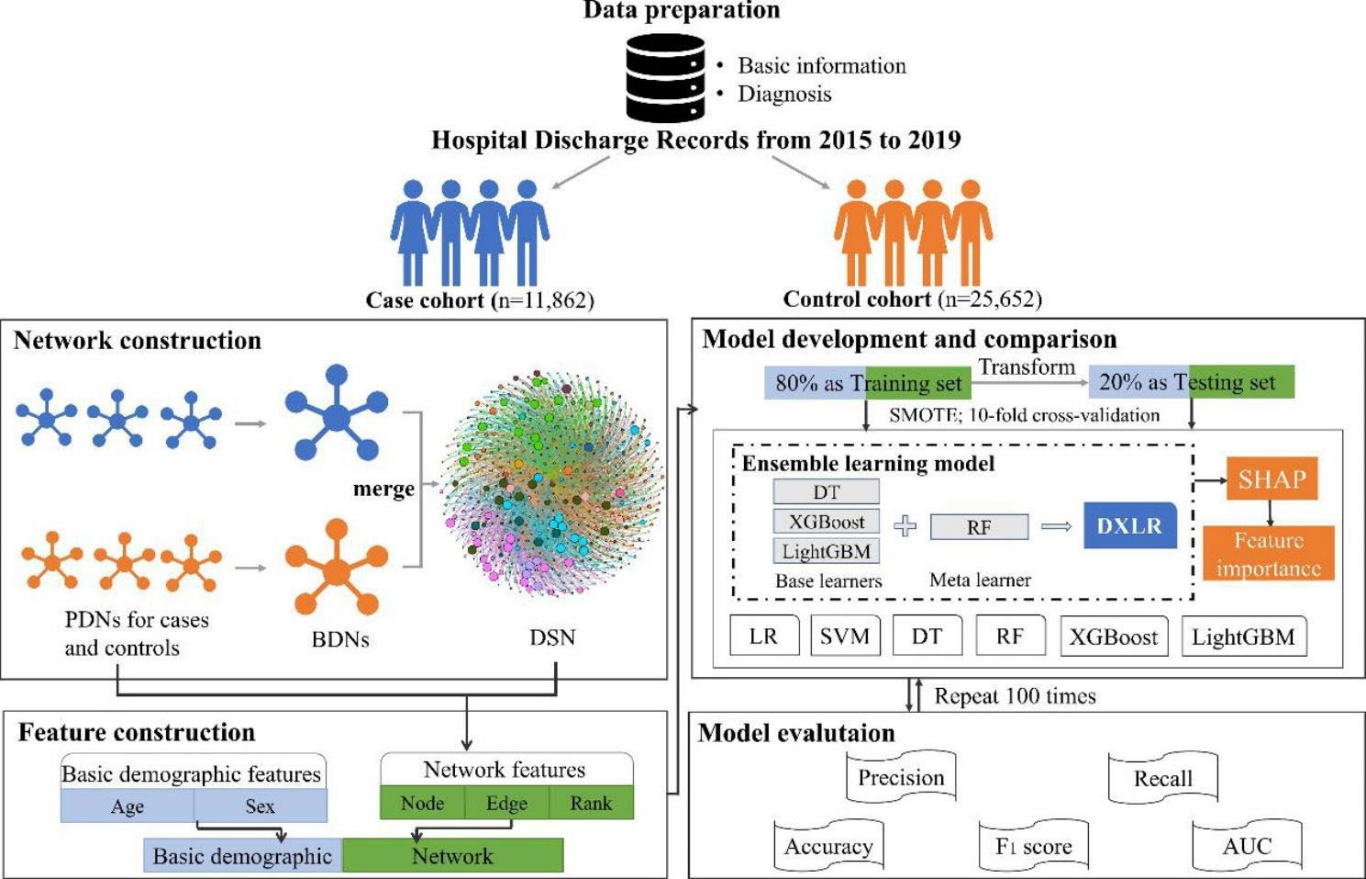
The overview of the framework for predicting HF risk in IHD patients. PDNs: Personal Disease Networks. BDNs: Baseline Disease Networks. DSN: Disease-Specific Network. DXLR: the two-stage ensemble machine learning model our study proposed

Figure 1 shows the architecture of the framework for predicting HF risk in patients with IHD. First, two cohorts, cases for patients first diagnosed with IHD and then with HF, and control IHD patients without HF, were established from the HDR dataset. Each patient’s HDR includes basic information, diagnosis history, admission and discharge time, etc. Then, three types of comorbidity networks, including personal disease network (PDN), baseline disease network (BDN), and disease-specific network (DSN), were constructed to identify patterns of patients’ diseases over time as well as the complex progression pattern from IHD to HF. Next, based on the DSN and PDN, three novel network features were generated to better characterize the progression patterns from IHD to HF. Moreover, to predict HF risk in IHD patients, while validating the effectiveness of the designed features, a stacking ensemble model and six traditional ML models were developed using different input features (basic demographic features and network features). Finally, the Shapley Addictive exPlanations (SHAP) [43] method was applied to analyze the feature importance of the proposed model.

### Data preparation

This is a large-scale, retrospective study based on anonymized HDRs collected from all the secondary and tertiary hospitals in Sichuan Province, China from January 1, 2015 to December 31, 2019. Each record contains de-identified codes, sex, age, admission and discharge times, and diagnosis information. Standard ICD-10 (International Classification of Diseases, 10th Revision) coding was used for all the disease diagnosis data. Furthermore, IHD and HF patients were identified by the first three digits of the ICD code (i.e., IHD: I20-I25 and HF: I50).

**Fig. 2:**
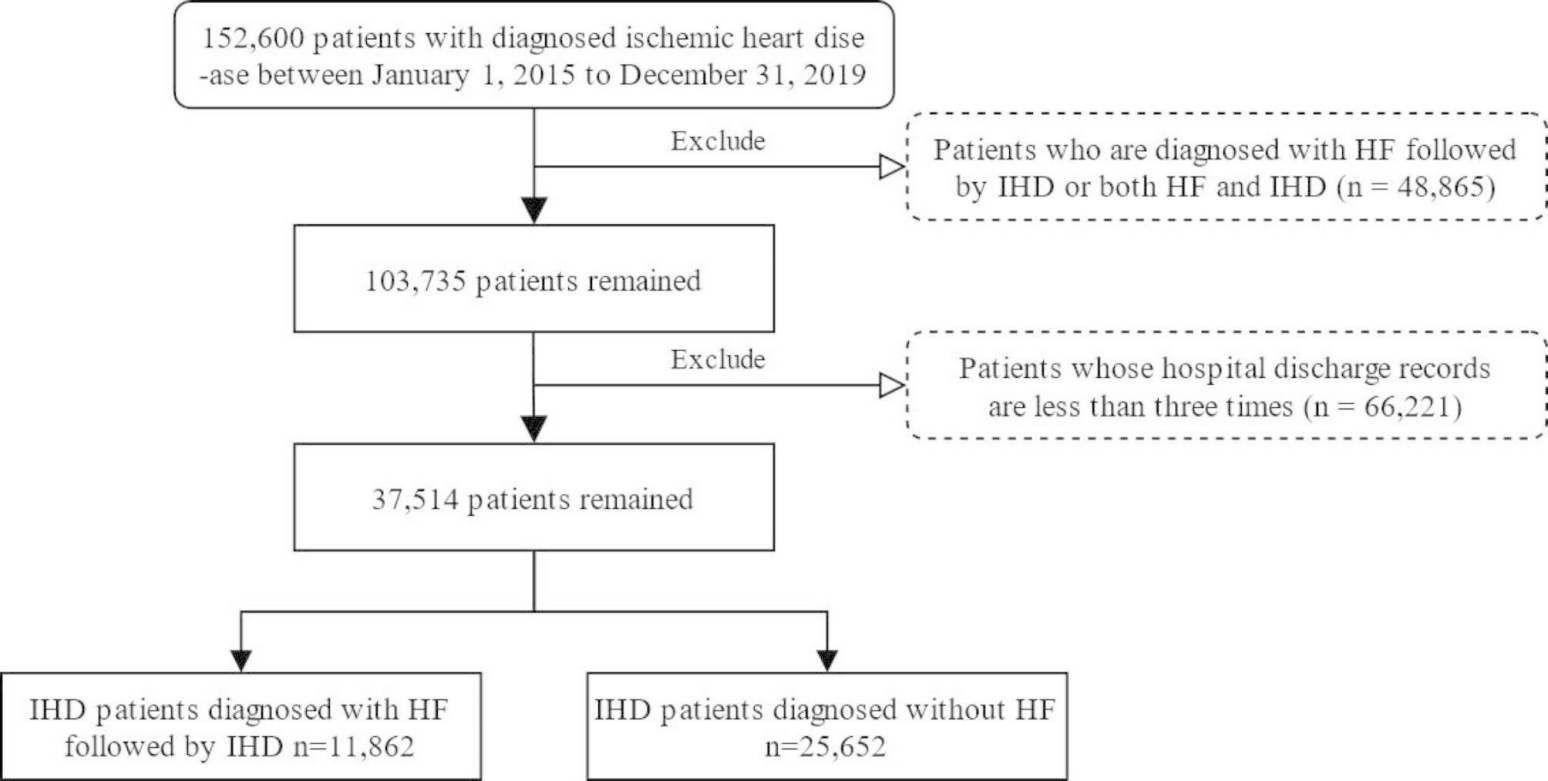
The extraction process for two patient cohorts

As shown in Fig. 2, two patient cohorts that met the selection criteria were selected from the database. After excluding data with missing values, invalid values, inconsistent data, and redundant data, HDR data for a total of 152,600 IHD patients were obtained. Patients firstly diagnosed with HF then followed by IHD or with both HF and IHD at the same time were excluded (n = 48,865). To identify the evolution pattern of disease over time, a more restrictive inclusion criterion was utilized, excluding patients with fewer than three hospital admissions (n = 66,221). Finally, a total of 37,514 IHD patients with mean ages of 70.2 ± 10.7 years were included in this study, of which 48.2% were male. Among them, 11,862 patients who were first diagnosed with IHD and then with HF during the study period served as the case group, while the other 25,652 patients without a diagnosis of HF after IHD diagnosis were served as the control group.

This study was approved by the Ethics Committee of Health Information Center of Sichuan Province. The requirement to obtain informed consent was waived because of the secondary nature of the de-identified data in the retrospective study design.

**Fig. 3:**
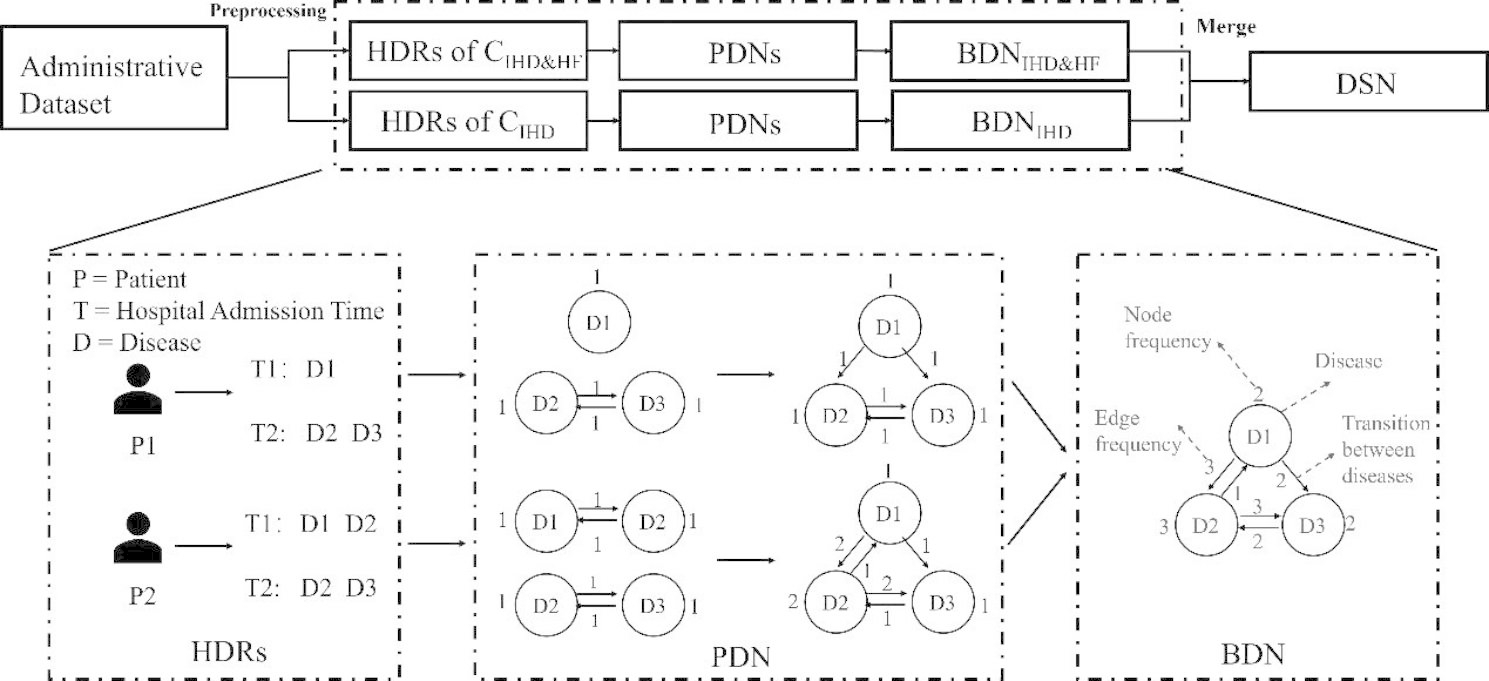
The process of constructing networks. Nodes represent diseases and directed edges represent the sequential relationship between diseases. HDRs: Hospital Discharge Records. C_IHD&HF_: Cohort of patients with IHD and HF. C_IHD_: Cohort of patients with IHD. PDNs: Personal Disease Networks. (adapted from [37]). BDN: Baseline Disease Network. DSN: Disease-Specific Network

### Network construction

The construction process of the disease networks is shown in Fig. 3. Three types of directed comorbidity networks were constructed using 65% of the entire dataset and the remaining 35% was used for modeling the ML models.

First, PDN was constructed for each patient in the two cohorts to describe the health trajectory of a patient during subsequent admissions over time [32]. In the PDN, the nodes, denoted as ${v}_{i} ({v}_{i} \in V\left(PDN\right))$, represent the diseases, and the edges, denoted as${{e}_{i} (e}_{i} \in E\left(PDN\right))$, represent the sequential relationships among diseases. The node weight, denoted as $freq\left({v}_{i}\right)$, indicates the prevalence of a patient’s disease in all admission events, and the edge weight, denoted as $freq\left({e}_{i}\right)$, indicates the number of times two diseases occurred during the same or consecutive admissions. Therefore, all nodes in PDN can be represented by a one-dimensional vector called disease vector. Each item in the vector represents a node ${v}_{i}$ and the corresponding value is the node weight $freq\left({v}_{i}\right)$. Similarly, all edges in the PDN can be represented by a two-dimensional matrix called disease adjacency matrix. Each element represents edge ${e}_{i}$ and the corresponding value is edge weight $freq\left({e}_{i}\right)$.

Next, to obtain the disease progression patterns of the patients in different cohorts, two BDNs (i.e., BDN_IHD & HF_ and BDN_IHD_) were constructed by merging the corresponding PDNs from the two cohorts. The nodes and edges of the BDN and the corresponding weights were calculated by summing the nodes and edges of all the PDNs in the same cohort.

**Fig. 4:**
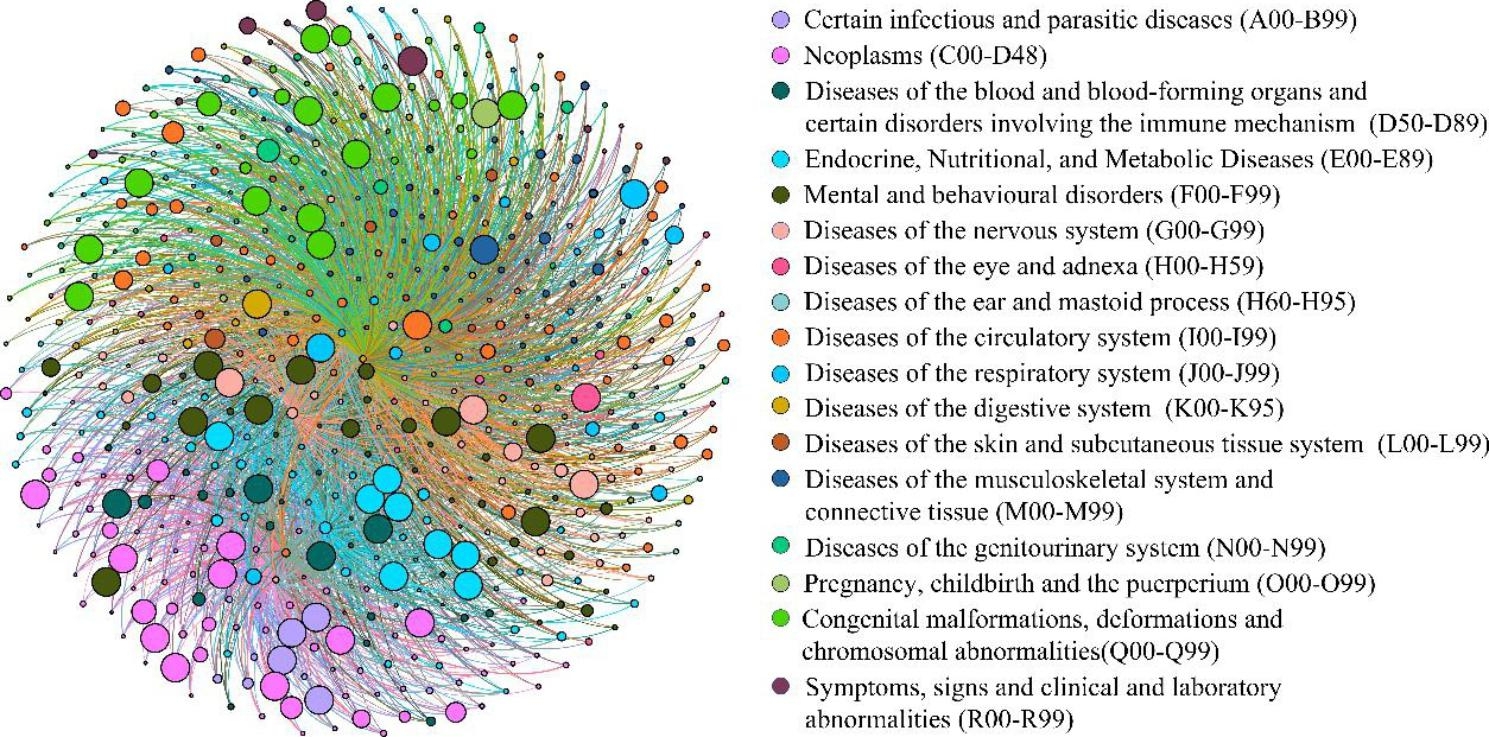
Visualization of the Disease-Specific Network. Nodes represent diseases and node sizes represent disease prevalence. Directed edges represent the sequence of occurrence between diseases and the frequency of two diseases that occurred during the same or consecutive admissions. Edges weighted less than 0.5 are hidden for simplicity

By considering the attribution theory [44], a final DSN was generated by combining BDN_IHD & HF_ and BDN_IHD_. To better characterize the disease patterns in the control group, all the control patients were included, which led to an unequal number of patients in the two cohorts. Then considering the impact of the number of patients included in the cohort on the weight of the disease network, the network weight was modified to the relative frequency. The DSN affords more weight to the chronic comorbidities which are more prevalent in BDN_IHD & HF_ than in BDN_IHD_. Moreover, it affords a low priority to the opposite conditions. The weight of the node and edge for DSN were calculated by determining their relative increments in BDN_IHD & HF_ compared to BDN_IHD_. The final DSN exhibited the specific disease trajectory of patients in cases [37]. Figure 4 displays the disease network visualization of DSN.

### Feature construction

As shown in Table 1, two types of features were extracted, including basic demographic features and network features.

#### Basic demographic features

Sex and age are risk factors for HF in IHD patients [6]. As shown in Table 1, these two basic characteristics were selected as the basic demographic features for modeling.

#### Network features

Three network features, including node score, edge score, and rank-based score were generated from the network to evaluate the HF risk in IHD patients, and to provide support from the network science perspective for early HF risk prediction.

##### Node score

Inspired by the node match score described in [41], a modified network feature, called the node score, was proposed based on the weighted disease vector similarity. To measuring the angular similarity between the disease vectors of the PDN and the DSN, the node score takes into account how closely the two vectors are positioned in the disease vector space. The closer the two vectors, the higher their similarity, and the higher the node score. The node score is a disease similarity-based metric that measures the relationship between PDN and DSN from the disease similarity perspective. A higher node score denotes that PDN has more similar diseases to DSN, specifically the diseases present in IHD patients with HF as compared to those without HF.

Mathematically, the node score for a patient (i.e., PDN) is defined as follows:

 1 ${F_{node}} = {{\mathop \sum \nolimits_{{v_i} \in V\left( {PDN} \right),{v_j} \in V\left( {DSN} \right),{v_i} = {v_j}} freq\left( {{v_i}} \right)*freq\left( {{v_j}} \right)} \over {\sqrt {\mathop \sum \nolimits_{{v_i} \in V\left( {PDN} \right)} freq{{\left( {{v_i}} \right)}^2}} *\sqrt {\mathop \sum \nolimits_{{v_j} \in V\left( {DSN} \right)} freq{{\left( {{v_j}} \right)}^2}} }}$

where ${v}_{i}$ is the vertex *i* (i.e., disease *i*) in the PDN, ${v}_{j}$ is the vertex *j* (i.e., disease *j*) in the DSN, and $freq\left(v\right)$ is the prevalence of a patient’s disease occurring in all admission events.

##### Edge score

Considering the evolutionary relationship between diseases over time, edge score was proposed as a metric based on the similarity of weighted disease vectors. The edges in the three constructed networks represent the temporal sequential relationship among diseases. The edge score was calculated based on the similarity of the edge vectors of the PDN and the DSN, which allowed for the characterization of the differences in the disease evolution paths between the two networks. A higher edge score suggests that the patient’s disease progression pattern is more similar to that of the DSN.

Mathematically, the edge score for a patient (i.e., PDN) is defined as follows:

 2 $${F_{edge}} = {{{\Sigma _{{e_i} \in E\left( {PDN} \right),{e_j} \in E\left( {DSN} \right),{e_i} = {e_j}}}freq\left( {{e_i}} \right)*freq\left( {{e_j}} \right)} \over {\sqrt {{\Sigma _{{e_i} \in E\left( {PDN} \right)}}freq{{\left( {{e_i}} \right)}^2}*} \sqrt {{\Sigma _{{e_j} \in E\left( {DSN} \right)}}freq{{\left( {{e_j}} \right)}^2}} }}$$

where ${e}_{i}$ is the edge *i* (i.e., disease pairs *i*) in the PDN, ${e}_{j}$ is the edge *j* (i.e., disease pairs *j*) in the DSN, and $freq\left(e\right)$ is the number of times the two diseases occurred during the same or consecutive admissions.

##### Rank-based score

The significance of disease in the PDN compared to the DSN was identified by applying the PageRank algorithm [45] to determine the disease importance of nodes within the DSN. The sum of the weighted disease importance of PDN nodes was calculated to obtain the rank-based score, which reflects the importance of diseases in the PDN with respect to the DSN. The score is based on the importance of nodes in the DSN’s network structure, i.e., it takes into account the importance of the disease nodes in the disease network as well as the relationship between the different diseases. A higher rank-based score for a patient’s PDN indicates the presence of more diseases in the PDN that are also found in the DSN. These diseases have a high prevalence in the PDN and are characterized by a high node importance in the DSN.

Mathematically, the rank-based score for a patient (i.e., PDN) is defined as follows:

3 $${F_{rank}} = {{{\Sigma _{{v_i} \in V\left( {PDN} \right),{v_j} \in V\left( {DSN} \right),{v_i} = {v_j}}}freq\left( {{v_i}} \right)*pg\left( {{v_j}} \right)} \over {\left| {V\left( {PDN} \right)} \right|}}$$

where ${v}_{i}$ is the vertex *i* (i.e., disease *i*) in the PDN, ${v}_{j}$ is the vertex *j* (i.e., disease j) in the DSN, $freq\left(v\right)$ is the prevalence of a patient’s disease occurring in all the admission events and $pg\left(v\right)$ is the PageRank value for vertex in the DSN, $\left|V\left(PDN\right)\right|$ is the total number of nodes (diseases) in the PDN.

**Table 1:** List of features considered in this study

Features	Descriptions	Number
**Basic demographic** **information**		**2**
Age	Patient’s age	1
Sex	Male or female	1
**Network**		**3**
Node	Network node cosine similarity match score extracted from PDN and DSN	1
Edge	Network edge cosine similarity match score extracted from PDN and DSN	1
Rank	Network node centrality match score extracted from PDN and DSN	1

### Ensemble learning model construction

Traditional ML methods are becoming increasingly popular in the field of disease prediction due to their excellent prediction abilities, while models generated from the same data among different ML algorithms have great heterogeneity [46]. Integrating various ML models might be a feasible way to produce a more powerful and robust model. This study developed a two-stage stacked ensemble learning model DXLR using network features and basic demographic features, which was comprised of three base learners (DT, XGBoost and Light Gradient Boosting Machine (LightGBM)) and a meta learner (RF).

**Fig. 5:**
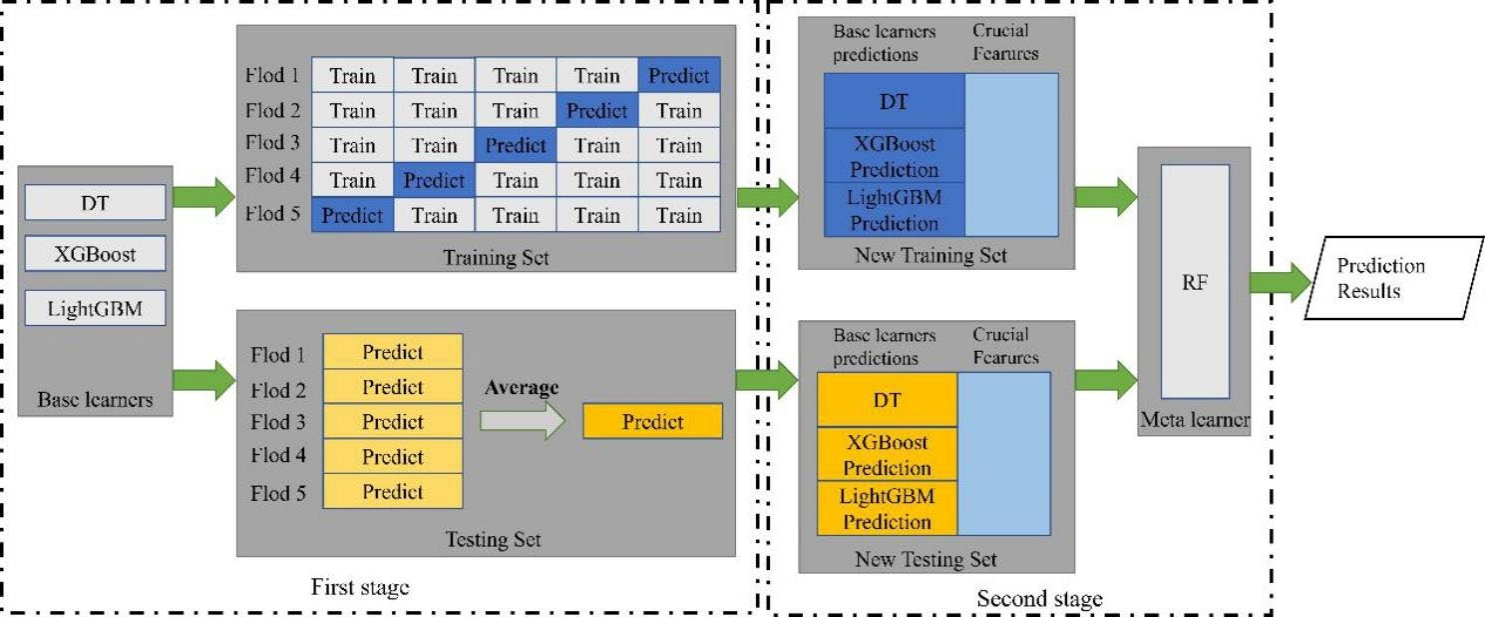
The overall framework of DXLR.

As shown in Fig. 5, in the first stage, five-fold cross-validation was performed for each of the models to generate a training set for the meta classifier. Among these folds, the base classifiers were used on four-folds, leaving one-fold for validation. Each base classifier output a new feature of the training set by merging the five validation folds and generated a new testing feature by averaging the five prediction results. Moreover, in the second stage, the most important feature of each basic learner was merged as crucial features to form the new training and testing set. The crucial features consisted of the most critical features in each base learner that have high feature importance for predicting HF risk in IHD patients.

### Model comparison and evaluation

To compared with DXLR model, six traditional ML classifiers, including LR [47], support vector machines (SVM) [48], DT [49], RF [50], XGBoost [51], and LightGBM [52], were developed. LR is a widely used classical linear model and has the advantage of fast convergence. As for SVM, the linear kernel was selected as the kernel function to improve the training efficiency of the model under millions of datasets. Before training the LR and SVM models, standard normalization was applied to the datasets. The DT, RF, XGBoost, and LightGBM models are tree-based ensemble models, which proved the nonlinear fitting ability and better ideas for improving the prediction model performance and robustness. A grid search strategy was adopted to determine the best model parameters.

A series of evaluation metrics, such as precision, recall, accuracy, and ${F}_{1}$ score, were used to evaluate the model performance. Furthermore, the area under the receiver operating characteristic curve (AUC) was obtained to compare the discrimination of the different ML models. To avoid data deviation caused by dataset partition, the dataset was randomly split into a training set (80%) and a testing set (20%) 100 times. The results were represented in the form of mean ± standard deviation. The six traditional ML models and the DXLR model were trained and validated on the training set through 10-fold cross-validation on each randomly divided sample set. Additionally, the Synthetic Minority Over-sampling Technique (SMOTE) [53] was used to avoid data imbalance.

4 $$Precision=\frac{TP}{TP+FP}$$

5 $$Recall=\frac{TP}{TP+FN}$$

6 $$Accuracy=\frac{TN+TP}{TN+TP+FN+FP}$$

7 $${F}_{1} score = \frac{2 \text{*} TP}{2\text{*}TP + FP + FN}$$

where *TP* denotes true positive, *FP* represents false positive, *TN* indicates true negative, *FN* denotes false negative.

To better understand the impact of different features on the results, the SHAP methods was applied to further extend and enhance the prediction results given by the DXLR model.

## Results

### Descriptive statistics

This study included 37,514 patients with IHD from January 1, 2015, to December 31, 2019. The basic characteristics of all IHD patients are shown in Table 2 and are classified by the presence or absence of subsequent HF diagnosis. Overall, 11,862 (31.6%) patients who were first diagnosed with IHD and then with HF during their hospitalization served as the case group, while the remaining 25,652 (68.4%) patients who were not diagnosed with HF served as the control group. The average age of the total patient group was 70.21 ± 10.74 years and 48.16% of the patients were male. The average age of the case group was 5.95 years higher than that of the control group (74.28 ± 9.55 vs. 68.33 ± 10.74; *P*-value < 0.001).

**Table 2:** Basic characteristics of the IHD patients

Characteristics	Total (n = 37,514)	Patients with HF (n = 11,862)	Patients without HF (n = 25,652)	*P*-value
Age(years)	70.21 ± 10.74	74.28 ± 9.55	68.33 ± 10.74	< 0.001
Sex				< 0.001
Male	18,067(48.16%)	5,870(49.48%)	12,197(47.55%)	
Female	19,447(51.84%)	5,992(50.51%)	13,455(52.45%)	

### Comparison of models

Table 3 displays the performance of the six traditional ML models and the DXLR model with both network features and basic demographic features considered. The results show that the proposed DXLR model exhibited higher performance and better stability on all evaluation metrics compared with other models. Although, XGBoost was the best performing model among the six traditional ML models, DXLR showed significantly improvement (*P*-value < 0.0001) in all metrics. As a result, the DXLR model was selected as the representative classifier for subsequent experiments.

**Table 3:** Comparison of the performance in six traditional models and the proposed DXLR model

Models	Metric [Mean ± SD]				
	Precision	Recall	Accuracy	F_1_ score	AUC
LR	0.538 ± 0.016	0.649 ± 0.016	0.712 ± 0.008	0.589 ± 0.013	0.766 ± 0.009
SVM	0.540 ± 0.016	0.644 ± 0.015	0.713 ± 0.008	0.587 ± 0.013	0.765 ± 0.009
DT	0.647 ± 0.015	0.827 ± 0.020	0.802 ± 0.007	0.726 ± 0.011	0.879 ± 0.006
RF	0.681 ± 0.015	0.833 ± 0.013	0.823 ± 0.007	0.749 ± 0.011	0.905 ± 0.005
XGBoost	0.714 ± 0.014	0.878 ± 0.012	0.850 ± 0.006	0.788 ± 0.010	0.928 ± 0.005
LightGBM	0.674 ± 0.015	0.836 ± 0.011	0.820 ± 0.007	0.746 ± 0.011	0.901 ± 0.005
**DXLR**	**0.723 ± 0.014**	**0.892 ± 0.012**	**0.857 ± 0.007**	**0.798 ± 0.010**	**0.934 ± 0.004**
*P*-value^a^	< 0.0001	< 0.0001	< 0.0001	< 0.0001	< 0.0001

^a^: *t*-test for the DXLR model and the best performing traditional model (XGBoost); The bold font is the best performing model

### Performance comparison of network features

The contributions of the three network features were further analyzed. As is shown in Table 4, the DXLR model with the rank-based score removed (i.e., using basic demographic features, node score, and edge score as input features) had the largest degradation in AUC performance. Meanwhile, the DXLR model with the node feature removed (AUC = 0.927) was marginally better than model with the edge feature removed (AUC = 0.923).

**Table 4:** Performance comparison of the DXLR model with removal of network feature separately

Method	Metric [Mean ± SD]				
	Precision	Recall	Accuracy	F_1_ score	AUC
DXLR	0.723 ± 0.014	0.892 ± 0.012	0.857 ± 0.007	0.798 ± 0.010	0.934 ± 0.004
−Node	0.699 ± 0.014	0.905 ± 0.010	0.846 ± 0.006	0.788 ± 0.009	0.927 ± 0.004
−Edge	0.681 ± 0.013	0.907 ± 0.012	0.836 ± 0.006	0.778 ± 0.009	0.923 ± 0.004
−Rank	0.492 ± 0.015	0.663 ± 0.019	0.676 ± 0.010	0.565 ± 0.012	0.737 ± 0.010

### Feature importance

**Fig. 6:**
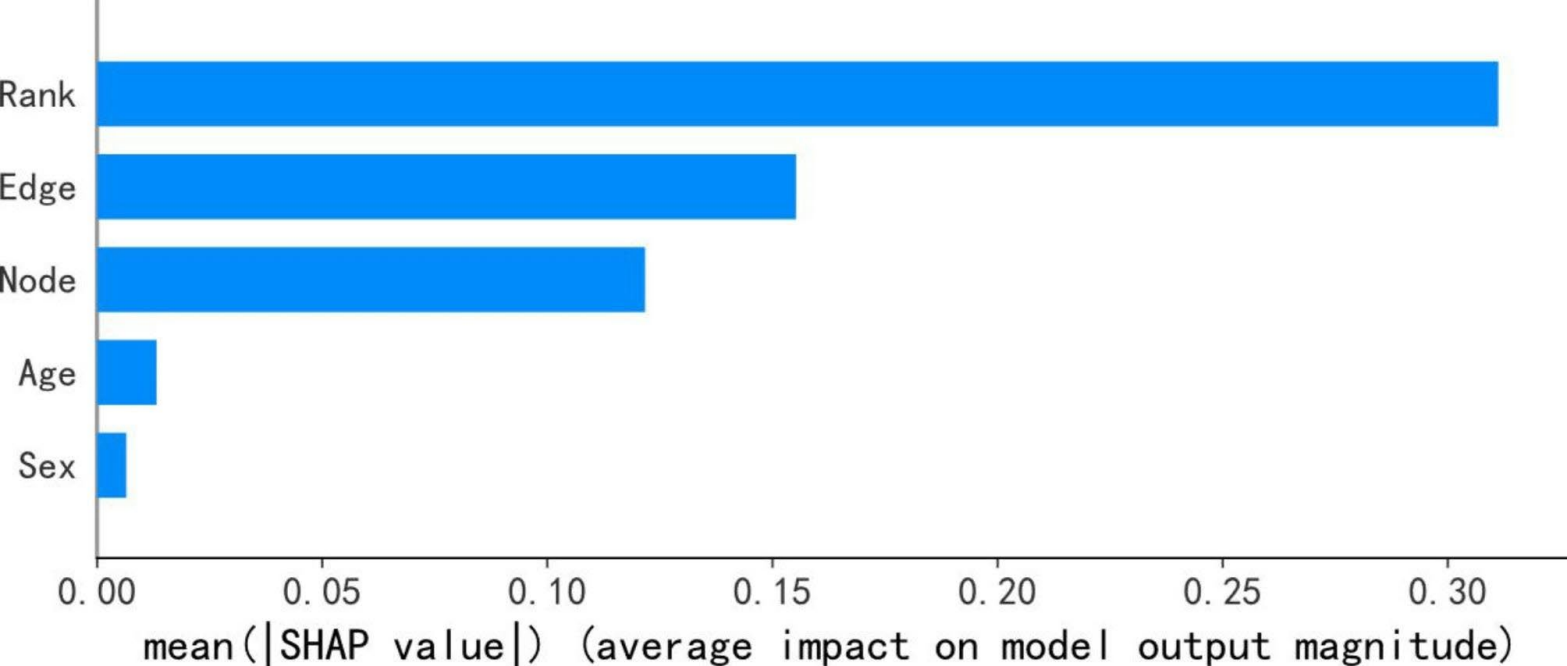
SHAP summary plot of the DXLR model. Average absolute impact of features on the final model output magnitude ordered by decreasing feature importance. Rank: the rank-based score. Edge: the edge score. Node: the node score

To visually explain the importance of different characteristics in the classification of the two groups of patients considered, SHAP was applied to illustrate how these features affect the performance of the DXLR model. Figure 6 shows all the features evaluated by the average absolute SHAP values. The feature ranking (y-axis) indicates the importance of the prediction model and the SHAP value (x-axis) is a uniform index reflecting the impact of a particular feature in the model. Overall, the absolute SHAP values of network features were considerably higher than those of the basic demographic features. The highest was the rank-based score (0.311), followed by the edge score (0.155) and the node score (0.122).

### Sensitivity analysis of the predictive model

To analyze the generalization performance of the DXLR model, the performance of our best classifier (with basic demographic features and network features) was compared on different subsets of patients. Table 5 lists the comparison results. The AUC and accuracy values of the predictive model for females were slightly higher (about 0.01) than those for males. The DXLR model showed no statistically significant difference in precision, recall, and F_1_ score values for different sex stratifications (*P*-value > 0.0001). In terms of the age groups, all the performance metrics exhibited statistically significant differences. The DXLR model showed the highest accuracy, precision, and AUC values for the 18–44 age group among the five age groups (0.798 ± 0.185, 0.960 ± 0.030, and 0.975 ± 0.044, respectively). While for the 80 + age group, the DXLR model had the highest recall and F_1_ score values and the lowest accuracy and AUC values.

**Table 5:** Performance in stratified subgroups

Subgroup	Metrics [Mean ± SD]				
	Precision	Recall	Accuracy	F_1_ score	AUC
**Sex**					
Male	0.716 ± 0.020	0.881 ± 0.017	0.848 ± 0.010	0.790 ± 0.015	0.926 ± 0.007
Female	0.722 ± 0.018	0.875 ± 0.019	0.857 ± 0.009	0.791 ± 0.014	0.932 ± 0.006
*P*-value^a^	0.0327	0.0277	< 0.0001	0.5533	< 0.0001
**Age groups**					
18–44	0.798 ± 0.185	0.901 ± 0.143	0.960 ± 0.030	0.832 ± 0.148	0.975 ± 0.044
45–59	0.685 ± 0.053	0.801 ± 0.060	0.919 ± 0.014	0.736 ± 0.042	0.953 ± 0.013
60–69	0.658 ± 0.034	0.809 ± 0.029	0.860 ± 0.013	0.725 ± 0.026	0.924 ± 0.010
70–79	0.725 ± 0.022	0.887 ± 0.018	0.838 ± 0.012	0.798 ± 0.015	0.921 ± 0.008
80+	0.757 ± 0.021	0.928 ± 0.017	0.816 ± 0.014	0.834 ± 0.015	0.894 ± 0.013
*P*-value^b^	< 0.0001	< 0.0001	< 0.0001	< 0.0001	< 0.0001

^a^: *t*-test; ^b^: one-way analysis of variance.

### Comparison with state-of-the-art study

Our proposed network features for predicting HF risk for IHD patients were also compared with those in a state-of-the-art study (three network features designed by Hossain [37]) under different classifiers. All the features were generated based on our dataset, and basic demographic features were sex and age. The DXLR model and RF (the best model in [37]) were selected as the models for feature comparison and verification. Table 6 verifies the superior performance of our features in all the evaluation metrics for both classifiers. The DXLR model using the network features proposed in this study outperformed the model using network features in [37], with an increase in precision by 30.7%, in recall by 37.4%, in accuracy by 18.7%, in F_1_ score by 33.7% and in AUC by 19.9%, respectively. Compared with the RF model using network features in [37], the precision, recall, accuracy, F_1_ score and AUC of the RF model using our network features increased by 28.0%, 18.3%, 15.9%, 23.6%, and 15.9%, respectively.

**Table 6:** Comparison of the classifier and features proposed in this study with the features proposed in previous study

Classifier	Network Features	Metrics [Mean ± SD]				
		Precision	Recall	Accuracy	F_1_ score	AUC
DXLR model	our study	0.723 ± 0.014	0.892 ± 0.012	0.857 ± 0.007	0.798 ± 0.010	0.934 ± 0.004
	[37]	0.553 ± 0.017	0.649 ± 0.021	0.722 ± 0.009	0.597 ± 0.013	0.779 ± 0.010
Random Forest	our study	0.681 ± 0.015	0.833 ± 0.013	0.823 ± 0.007	0.749 ± 0.011	0.905 ± 0.005
	[37]	0.532 ± 0.016	0.704 ± 0.016	0.710 ± 0.009	0.606 ± 0.013	0.781 ± 0.010

## Discussion

This study proposes an approach to predict high-risk groups for HF among IHD patients using routinely collected administrative data. By integrating network analytics with ensemble learning, our approach is able to extract disease patterns hidden in administrative data and identify patients at high risk that may benefit from screening and a preventive strategy. This method could be used in other regions where large administrative datasets can be linked at the individual person level to help health authorities identify high-risk groups and formulate targeted policies to better guide individuals, thus reducing the risk of illness.

In this study, the performance of the DXLR model was compared with six traditional models, and the results showed that the DXLR model outperformed all the other models. Among the traditional models, tree-based models performed relatively better compared to LR and SVM, with XGBoost exhibiting the best performance. However, the proposed DXLR model performed significantly better (*P*-value < 0.0001) than the XGBoost model in all metrics, achieving a precision of 0.723, a recall of 0.892, an accuracy of 0.857, an F_1_ score of 0.798 and an AUC of 0.934. This performance improvement highlights the effectiveness of our DXLR model, which combines the strengths of multiple models to achieve higher prediction accuracy.

Although there were some variances in performances across subgroups, such as a slightly lower AUC in male patients and decreasing AUC with increasing age, our proposed DXLR model still demonstrated a strong and stable predictive ability. These differences in performance could be due to their differences in comorbidity burden and in complex comorbidity relationships in male and older IHD patients [7]. Nevertheless, our model provides a promising tool for identifying high risk groups for HF in diverse IHD patient populations.

The comparison of the contributions of the three network features revealed that the removal of the rank-based score led to the most significant drop in AUC (0.197), followed by a 0.011 and 0.007 decrease in AUC when the edge score and node score were removed, respectively. Furthermore, SHAP was applied to validate the contribution of the three network features to the prediction results of the DXLR model. The rank-based score yielded the highest SHAP value score, with about twice as important as the edge and the node score. These results suggest that the rank-based score is the most critical network feature for predicting HF risk in IHD patients. One possible explanation for this finding is that the rank-based score captures the relative importance of diseases within the DSN network. A higher number of specific diseases in a patient’s PDN that are important for the disease progression patterns from IHD to HF in the DSN, may increase the likelihood of developing HF. Additionally, the edge score reflect the similarity of disease progression trajectories between patients, while the node score capture the overall disease burden of patients. The combination of these network features provides a more comprehensive characterization of the risk of HF in IHD patients and helps to identify high risk patients that could benefit from early screening and prevention strategies.

The three network features proposed by our study exhibit better prediction performance than the network features designed in a state-of-the-art study [37] under the same model and basic demographic features. As shown in Table 6, compared with the features designed by Hossain et al. [37], our network features captured the complex comorbid and progressive relationship between IHD and HF, improving the predictive metrics of the DXLR model by 0.135–0.243. Compared with the best-performing RF model in [37], the precision and F_1_ score improved by 0.149 and 0.143, respectively. These network features were used to measure the propensity of patients to progress from IHD to HF with node score and edge score characterized the disease propensity and disease pair progression propensity of IHD patients by measuring the similarity of disease vectors and disease pair vectors of PDN and DSN, respectively. The rank-based score portrays the disease-weighted propensity of IHD patients by measuring the similarity of the weighted disease vectors of PDN and DSN. Therefore, models using our proposed network features performed better than those in previous work.

Our study has several limitations. First, the model this study proposed is only suitable for early risk prediction, not for clinical auxiliary diagnosis. Second, our network features were proposed based on the association of disease pairs or the progressive relationship of disease pairs. The prediction accuracy could be further improved by considering the patient-to-patient similarity inherent in the administrative dataset [38, 54]. In addition, graph neural networks (GNNs) are increasingly popular for learning network-based tasks [55, 56]. Future research is recommended to incorporate GNN-based algorithms to better utilize the network and improve its predictive performance.

## Conclusions

This study proposed an approach to predict risk of HF in patients with IHD by integrating network analytics with ensemble learning. Experimental results showed our proposed DXLR model outperformed the other traditional ML models. Further experiments also demonstrated our proposed network features exhibited better performance on the same data and model compared with the features created by the state-of-the-art study. These results highlight the potential value of network-based ML in disease risk prediction field using administrative data.

## Data Availability

The data that support the findings of this study are available from Health Information Center of Sichuan Province but restrictions apply to the availability of these data, which were used under license for the current study, and so are not publicly available. Data are however available from the corresponding author upon reasonable request and with permission of the Health Information Center of Sichuan Province.

## Abbreviations

IHD: Ischemic heart disease
HF: Heart failure
CVD: Cardiovascular disease
PDN: Personal disease network
BDN: Baseline disease network
DSN: Disease-specific network
SHAP: Shapley addictive explanations
XGBoost: Extreme gradient boosting
DT: Decision tree
LightGBM: Light gradient boosting machine
RF: Random forest
LR: Logistic regression
SVM: Support vector machine
